# Editorial: Alzheimer's Disease and the Fornix

**DOI:** 10.3389/fnagi.2016.00149

**Published:** 2016-06-23

**Authors:** Kenichi Oishi, Constantine G. Lyketsos

**Affiliations:** ^1^The Russell H. Morgan Department of Radiology and Radiological Sciences, Johns Hopkins UniversityBaltimore, MD, USA; ^2^Department of Psychiatry and Behavioral Sciences, Johns Hopkins UniversityBaltimore, MD, USA

**Keywords:** Alzheimer's disease, fornix, limbic, diffusion tensor imaging, normal aging, memory, cognition, mild cognitive impairment

The fornix is a white matter bundle that connects the hippocampus with other limbic structures. It appears in the literature as early as 1543 in a historical publication of *De Humani Corporis Fabrica* by Andreas Vesalius (Swanson, 2014). The fornix is important for episodic memory recall (Tsivilis et al., 2008), which is impaired in Alzheimer's disease (AD). Alterations in the fornix were first observed in post-mortem AD brains (Hopper and Vogel, 1976). This volume focuses on the role of the fornix, and other limbic fibers, in the disease mechanisms of AD with some attention to how this might be applied in clinical practice.

The observation of limbic fibers *in vivo* forms a basis for understanding normal anatomy and alterations caused by various diseases. Mori and Aggarwal were able to observe the fornix, cingulum, and stria terminalis in mice and humans, using T1-weighted and diffusion tensor imaging.

In adult human brains, the limbic fibers are known to connect the structures of the default mode network (DMN), but the development of these fibers is less well understood. Yu et al. demonstrated that the developmental curve of DTI-derived measures of fornix integrity appear logarithmic, with rapid changes until 2 years of age followed by slow changes until 25 years. Development of the cingulate cingulum is disproportionally rapid during this period, but development of the hippocampal cingulum and the fornix is proportional. Notably, the functional and anatomic connectivity of the DMN is already established in the early postnatal period.

The fornix is among the white matter structures that mature early during development. Douet and Chang reviewed changes in DTI measures in the fornix during development and aging. Development of the fornix peaks in late adolescence, followed by pruning and then degeneration. Fractional anisotropy (FA) values correlate with cognitive performance in various age groups, including children, young adults, and the elderly. Correlations are seen in various brain diseases including schizophrenia, multiple sclerosis, Parkinson's, and epilepsy.

Normal aging affects the anatomy of the fornix. Kantarci proposed a hippocampus-fornix axis in which microstructural alterations in both hippocampus and fornix affect each other. In AD, it is likely that neuronal damage in hippocampus and axonal damage in fornix affect each other, but alterations in the fornix in normal aging are likely the consequence of age-related, non-specific axonal and myelin damage.

Less is known about the relationship between alterations in fornix and functional connectivity. Kehoe et al. investigated whether diffusivity in fornix is related to functional connectivity between thalamus and hippocampus. Several diffusivity measures were correlated with functional connectivity among cognitively normal elderly, but this correlation was not seen in individuals with amnestic mild cognitive impairment (MCI). This suggests that the pathological processes of amnestic MCI mitigate the structural-functional relationship that is normally seen.

Tract-based spatial statistics (TBSS) are commonly used to analyze neuroanatomical alterations related to AD. Acosta-Cabronero and Nestor reviewed AD studies that applied TBSS. In early AD, increases in the first eigenvalue were identified in fornix, parahippocampal white matter, and anterior thalamus. The authors emphasized the importance of technological factors that affect results of clinical DTI studies. These include the basis of diffusion-weighted signals and tensor calculation, the imaging parameters, the post-processing methodology, the subject cohort, multi-center study designs, and inclusion criteria. This review is quite helpful to investigators who plan to study DTI of AD or other diseases.

Nowrangi and Rosenberg summarized DTI-derived scalar measures as correlates of cognitive functions or Aβ deposition in AD, as predictors of future conversion from MCI to AD, and as potential targets for deep brain stimulation. Fletcher et al. applied these markers to a cohort of cognitively normal elderly to predict later occurrence of brain atrophy. Reduced FA was used as a marker for white matter alterations in various structures, including fornix, genu, splenium of the corpus callosum, and anterior and posterior cingulum. Volume reduction was used to evaluate neurodegeneration in the hippocampus and in the ventral and dorsal entorhinal cortices. Fornix FA was the most sensitive marker among structures investigated in predicting future brain atrophy typically seen in AD.

One direction for the future in neuroimaging studies is the application of research to the clinical arena, in which prediction is an important theme. Oishi and Lyketsos reviewed DTI analysis methods reported to detect anatomical abnormalities in the AD brain, especially fornix, and discussed the potential for the early diagnosis, prediction of cognitive worsening, and therapeutic targets.

Disease specificity is often an issue in clinical image reading. Although alterations in the fornix are often seen in AD, such alterations are also seen in other diseases, such as temporal lobe epilepsy. Alexander et al. investigated the relationship between limbic fiber integrity and cognitive function using DTI in patients with temporal lobe epilepsy. They identified a correlation between FA of the left fornix and processing speed, but not between T2 of the hippocampus and processing speed. This suggested that the relation between fornix injury and functional decline is not disease-specific, but rather, the result of injury in a neuronal network with specific neuronal functions.

In summary, the 10 articles included in this volume cover anatomy, development, aging, disease, and functional correlations or clinical significance, which are informative for readers who plan to investigate the fornix in AD or other diseases.

## Author contributions

KO wrote preliminary draft and CL finalized the manuscript.

### Conflict of interest statement

The authors declare that the research was conducted in the absence of any commercial or financial relationships that could be construed as a potential conflict of interest.
